# Association between the Dietary Inflammatory Index and Gastric Disease Risk: Findings from a Korean Population-Based Cohort Study

**DOI:** 10.3390/nu14132662

**Published:** 2022-06-27

**Authors:** Sundara Raj Sreeja, Trong-Dat Le, Bang Wool Eom, Seung Hyun Oh, Nitin Shivappa, James R. Hebert, Mi Kyung Kim

**Affiliations:** 1 Department of Cancer Epidemiology, National Cancer Center, Ilsandong-gu, Goyang 10408, Korea; ssreejaraj@gmail.com; 2 Department of Cancer Biomedical Science, Graduate School of Cancer Science and Policy, National Cancer Center, Goyang 10408, Korea; datle90@ncc.re.kr; 3 Center for Gastric Cancer, National Cancer Center, Ilsandong-gu, Goyang 10408, Korea; kneeling79@ncc.re.kr; 4 College of Pharmacy, Gachon University, Incheon 21936, Korea; eyeball@hanmail.net; 5 Cancer Prevention and Control Program, University of South Carolina, Columbia, SC 29208, USA; shivappa@mailbox.sc.edu (N.S.); jhebert@mailbox.sc.edu (J.R.H.); 6 Department of Epidemiology and Biostatistics, Arnold School of Public Health, University of South Carolina, Columbia, SC 29208, USA; 7 Department of Nutrition, Connecting Health Innovations LLC, Columbia, SC 29201, USA

**Keywords:** dietary inflammatory index, gastric diseases, pro-inflammatory diet, anti-inflammatory diet, gastritis, ulcer

## Abstract

Evidence suggests that diets with high pro-inflammatory potential may play a substantial role in the origin of gastric inflammation. This study aimed to examine the association between the energy-adjusted dietary inflammatory index (E-DII^TM^) and gastric diseases at baseline and after a mean follow-up of 7.4 years in a Korean population. A total of 144,196 participants from the Korean Genome and Epidemiology Study_Health Examination (KoGES_HEXA) cohort were included. E-DII scores were computed using a validated semi-quantitative food frequency questionnaire. Multivariate logistic regression and Cox proportional hazards regression were used to assess the association between the E-DII and gastric disease risk. In the prospective analysis, the risk of developing gastric disease was significantly increased among individuals in the highest quartile of E-DII compared to those in the lowest quartile (HR_quartile4vs1_ = 1.22; 95% CI = 1.08–1.38). Prospective analysis also showed an increased risk in the incidence of gastritis (HR_quartile4vs1_ = 1.19; 95% CI = 1.04–1.37), gastric ulcers (HR_quartile4vs1_ = 1.47; 95% CI = 1.16–1.85), and gastric and duodenal ulcers (HR_quartile4vs1_ = 1.46; 95% CI = 1.17–1.81) in the highest E-DII quartile compared to the lowest quartile. In the cross-sectional analysis, the E-DII score was not associated with the risk of gastric disease. Our results suggest that a pro-inflammatory diet, indicated by high E-DII scores, is prospectively associated with an increased risk of gastric diseases. These results highlight the significance of an anti-inflammatory diet in lowering the risk of gastric disease risk in the general population.

## 1. Introduction

The acute inflammatory response is essential for ensuring that immune cells protect the body from bacteria, viruses, toxins, and spontaneous mutations and for promoting tissue repair and recovery from injury or infection [1,2]. Invasion by microbes and tissue damage due to injury can promote acute inflammation that lasts for a short period [3]. Chronic inflammation can last for several months, years, or even decades, depending on the cause of the injury and its repair mechanism [4]. Gastric diseases associated with the stomach include gastritis and ulcers [5]. Gastritis is a chronic condition characterized by inflammation of the mucous membrane in the stomach, where gastric cells are replaced by intestinal-type epithelium and fibrous tissue [6,7]. The common mechanism in gastric disease etiology is the disproportion between mucosal deformation and its repair mechanisms in the stomach [8]. Ulcers, or slow-healing sores, are chronic inflammatory states that develop in the lining of the stomach or upper part of the small intestine [9]. Chronic inflammation is implicated in severe medical conditions such as cancer and other chronic diseases [10,11,12,13,14].

Helicobacter *pylori* is considered a significant causal factor and a class 1 carcinogenic agent in the pathogenesis of gastric diseases [15]. H. *pylori* colonization leads to chronic gastritis, which progresses to atrophic gastritis, intestinal metaplasia, and eventually gastric cancer [16]. Other risk factors associated with gastric inflammation include alcoholism, smoking, non-steroidal anti-inflammatory drug use, tobacco use, stress, surgical procedures, irradiation in the stomach, autoimmune disorders, and systemic viral infections [17,18,19,20]. Prolonged use of non-steroidal anti-inflammatory drugs increases mucosal barrier permeability, reduces mucosal blood flow, and causes ulceration and inflammation in the gastric mucosal lining [21].

Previous studies have shown that diet plays an important role in the etiology of chronic inflammation of the gastric mucosa [22,23,24,25,26,27,28,29,30,31]. A high-fat diet impairs the gastrointestinal tract by producing bile acids, resulting in the expression of pro-inflammatory enzymes and cytokines that contribute to the development of insulin resistance [22]. Resulting in inflammation, increased oxidative damage due to high levels of reactive oxygen species and increased apoptosis destroys the gastric mucosa [23]. Sugars in carbonated drinks reduce esophageal sphincter pressure and increase gastroesophageal pressure, which causes irritation in the mucosal lining and alters the gut microbiota [24]. Spicy foods provoke irritation of the mucous membrane by elevating the levels of gastric secretion, and inflammation of the stomach lining [25]. However, in the long term, these spicy foods reduce chronic, systemic, and tissue-specific simmering inflammation [32]. In addition, excessive salt intake increases epithelial damage, followed by parietal cell loss and severe *H. pylori* colonization, and has been associated with an increased risk of atrophic gastritis and with intestinal metaplasia [26,27].

No previous studies have presented epidemiological evidence on the association between the energy-adjusted dietary inflammatory index (E-DII^TM^) and gastric disease risk. This study aimed to examine the association between the inflammatory potential of diet as estimated by the E-DII, and the incidence of gastric diseases at baseline and after a follow-up period. The study hypothesis was that high E-DII scores corresponding to a pro-inflammatory diet increase the risk of gastric diseases, including gastritis and ulcers.

## 2. Materials and Methods

### 2.1. Study Population and Data Collection

The Korean Genome and Epidemiology Study_Health Examination (KoGES_HEXA) is a large prospective cohort study that explores the relationship between genetic, environmental, and lifestyle factors in chronic diseases such as hypertension, metabolic syndrome, obesity, type 2 diabetes, and cardiovascular disease among Koreans. The present study used data from the KoGES-HEXA study to investigate the association between the energy-adjusted dietary inflammatory index (E-DII^TM^) and gastric diseases. All study subjects deliberately responded to the baseline survey through on-site invitations, letters, telephone calls, campaigns, and community conferences. Detailed information on KoGES can be found elsewhere [33].

**Figure 1:**
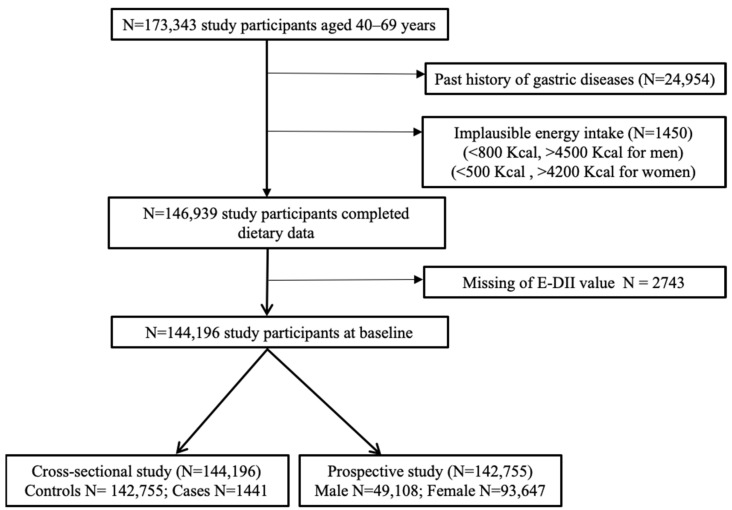
Flowchart of study subjects included in this study from the KoGES_HEXA cohort.

The KoGES health examination study included 173,343 participants aged 40–69 years, recruited from 38 health examination centers and hospitals located in 8 regions of Korea [34]. At baseline, the study participants were enrolled based on a standardized study protocol authorized by the Ethics Committee of the Korean Health and Genomic Study of the Korean National Institute of Health and Institutional Review Boards. Informed consent was obtained from all participants prior to their participation in the study. The Institutional Review Board of the National Cancer Center in Korea approved all methods used in the present study, and all relevant guidelines and regulations were followed (IRB No. NCC2018-0164). Among the original 173,343 participants, we excluded subjects who had a history of gastric diseases (*n* = 24,954), extreme calorie intake [males <800 Kcal or >4500 Kcal; females <500 Kcal or >4200 Kcal; (*n* = 1450)], or insufficient data to compute E-DII scores (*n* = 2743). After exclusion, data from 144,196 study subjects were included in the final analysis. After a mean follow-up of 7.4 years, 1571, 600, and 102 cases of gastritis, gastric ulcers, and duodenal ulcers were identified, respectively. The incidence of inflammatory diseases was self-reported by the study participants after diagnosis by a medical doctor (Figure 1).

### 2.2. Statistical Analysis

To examine the association between the E-DII and gastric disease, the E-DII was categorized into quartiles. Continuous variables were expressed as means with standard deviations, and categorical variables were expressed as frequencies with percentages. An analysis of variance was used for continuous variables with *p* values representing the likelihood of obtaining this result on the assumption that the null hypothesis of no effect was true, while the chi-square test was applied to categorical variables. Logistic regression analysis was performed to analyze the association between the E-DII score and gastric disease at baseline. The association between the E-DII and gastric disease incidence was analyzed using multivariate Cox proportional hazards models. The models were adjusted for sex, age (categorical), educational status (categorical), smoking (categorical), drinking (categorical), physical activity (categorical), energy (continuous), and body mass index (BMI) (continuous). The odds ratios (ORs) and hazard ratios (HRs) were calculated with 95% confidence intervals (CIs) for logistic regression and the Cox proportional hazards model, respectively. The *p*-value for the interaction between E-DII and sex was calculated from the likelihood ratio test comparing the multivariate-adjusted model with and without the product terms of E-DII and sex. The *p*-value for the trend was computed by assigning the median value for each quartile and treating it as a continuous variable. Subgroup analysis was performed by stratifying the total population according to smoking, drinking, physical activity, BMI, age, and menopausal status. Cubic spline regression was performed to assess the dose-response association between the E-DII score and the risk of gastric disease. A Cox proportional hazards model was used to examine the association between each food and nutrient component and gastric diseases. Results with *p* values of <0.05 were considered statistically significant. All statistical analyses were performed using R version 4.1.1. Additional details regarding the materials and methods can be found in the Supplementary Data.

## 3. Results

### 3.1. Baseline Characteristics of the Study Subjects

Table 1 shows the distribution of the demographic characteristics of the 144,196 study participants according to the E-DII quartiles. As the E-DII score increased, the mean energy intake decreased (*p* < 0.0001). The higher E-DII groups were more likely to be older and overweight but have a lower level of education, be less physically active, experience menopause, and have a family history of cancer (*p* < 0.0001). Moreover, the percentage of never smokers and drinkers decreased as the E-DII increased (*p* < 0.0001). A similar trend was observed in females, while the opposite was observed in males.

Table S1 shows the distribution of food and nutrient parameters across E-DII quartiles in the KoGES_HEXA cohort. Significant differences were observed for cereals, nuts, eggs, spinach, carrot, pork, chicken, beef, milk, coffee, and carbonated beverages.

### 3.2. E-DII and Prevalent Gastric Diseases at Baseline

In cross-sectional analyses of baseline data there was no significant association with any gastric diseases found in the highest compared to the lowest E-DII quartile in the total population (OR_quartile4vs1_ = 1.09; 95% CI = 0.94–1.26; *p*-trend = 0.20), in males (OR_quartile4vs1_ = 1.01; 95% CI = 0.77–1.32; *p*-trend = 0.14), or in females (OR_quartile4vs1_ = 1.13; 95% CI = 0.95–1.34; *p*-trend = 0.19) (Table 2). Similar results were observed for gastritis, gastric ulcers, duodenal ulcers, and gastric and duodenal ulcers.

### 3.3. E-DII and Incident Gastric Diseases

During a follow-up of 7.4 years, there were 1571 incident cases of gastritis (412 males and 1159 females), 600 gastric ulcers (207 males and 393 females), and 102 duodenal ulcers (38 males and 64 females). Overall, a higher risk of incident gastric diseases was obtained in the highest E-DII quartile (HR_quartile4vs1_ = 1.22; 95% CI = 1.08–1.38; *p*-trend = 0.01) compared to the lowest quartile (Table 2). Females in the highest quartile also showed a 27% higher risk of gastric diseases (HR_quartile4vs1_ = 1.27; 95% CI = 1.08–1.45; *p*-trend = 0.01) than those in the lowest quartile. Similar results were observed for gastritis incidence in the total population (HR_quartile4vs1_ = 1.19; 95% CI = 1.04–1.37; *p*-trend = 0.01) and in females (HR_quartile4vs1_ = 1.19; 95% CI = 1.02–1.40; *p*-trend = 0.03). Moreover, prospective analysis showed a significant association between a pro-inflammatory diet and a higher risk of developing gastric ulcers (HR_quartile4vs1_ = 1.47; 95% C I = 1.16–1.85; *p*-trend = 0.01) and gastric and duodenal ulcers (HR_quartile4vs1_ = 1.46; 95% CI = 1.17–1.81; *p*-trend = 0.01) compared to that of an anti-inflammatory diet. Females in the highest E-DII quartile were also observed to have a 68% higher risk of developing gastric ulcers (HR_quartile4vs1_ = 1.68; 95% CI = 1.25–2.25; *p*-trend = 0.01) and a 65% higher risk of developing gastric and duodenal ulcers (HR_quartile4vs1_ = 1.65; 95% CI = 1.26–2.17; *p*-trend = 0.01) in the highest E-DII quartile compared to the lowest quartile. No significant association was observed with the risk of duodenal ulcers.

**Figure 2:**
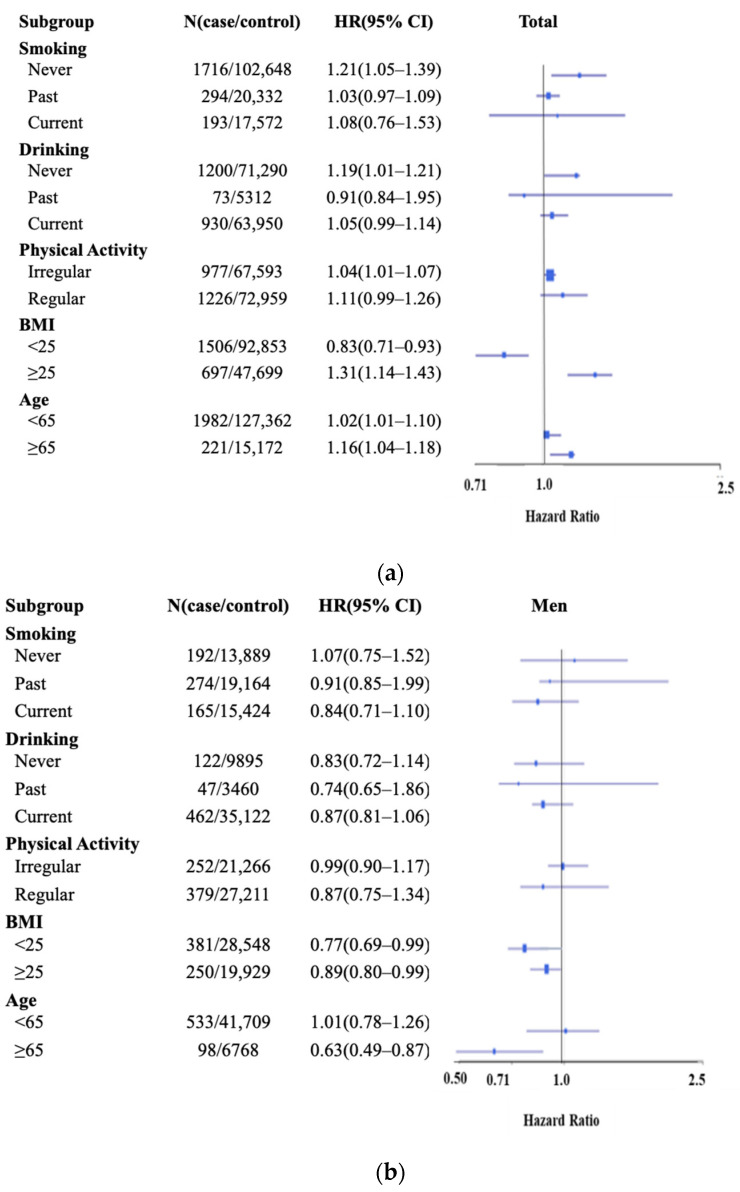

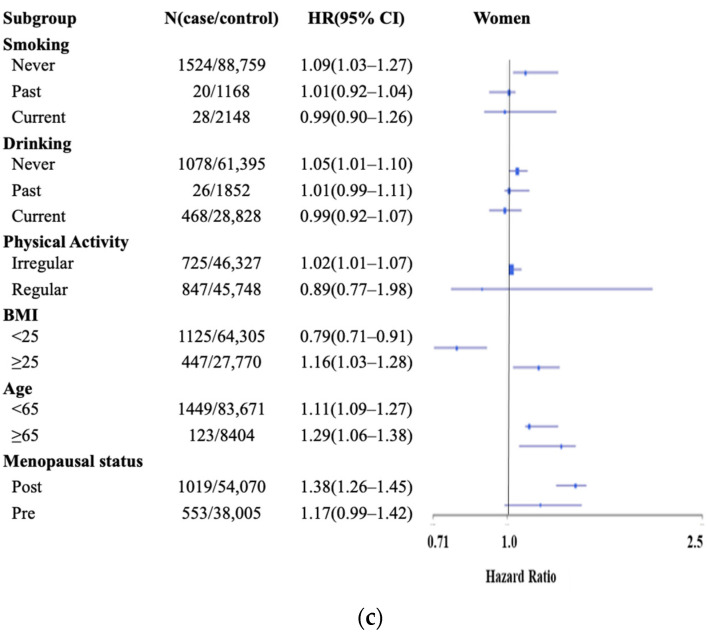
Subgroup analysis of the KoGES_HEXA among total (**a**), men (**b**), and women (**c**) by smoking, drinking, physical activity, BMI, age, and menopausal status (only for women) for gastric disease (gastritis, gastric ulcer, and duodenal ulcer) risk. HRs, hazard ratios; 95% CIs, 95% confidence intervals.

Figure 2 shows the subgroup analysis by smoking, drinking, physical activity, BMI, age, and menopausal status for the association between the E-DII and gastric disease risk. In the total population, a significant association with increased risk of gastric diseases was seen in the never-smoker group, never-drinker group, high-BMI group, both physical activity groups, and age groups, while a decreased risk was seen in the low-BMI group. The same findings were reported in females, except for the regular physical activity group, in which the association was not significant. In males, a significant association was observed only in the higher BMI group and older males.

As shown in Table S2, the highest quartile of the E-DII in postmenopausal females showed a significantly increased risk of gastritis, gastric ulcers, and gastric and duodenal ulcers compared to the lowest quartile. Premenopausal females showed no significant association between the E-DII and gastric disease. Table S3 shows that study subjects over 65 years of age reported a significant association with incident gastritis (HR_quartile4vs1_ = 1.07; 95% CI = 1.03–1.24; *p*-trend = 0.01), gastric ulcers (HR_quartile4vs1_ = 1.21; 95% CI = 1.07–1.34; *p*-trend = 0.01), and gastric and duodenal ulcers (HR_quartile4vs1_ = 1.10; 95% CI = 1.02–1.09; *p*-trend = 0.01).

**Figure 3:**
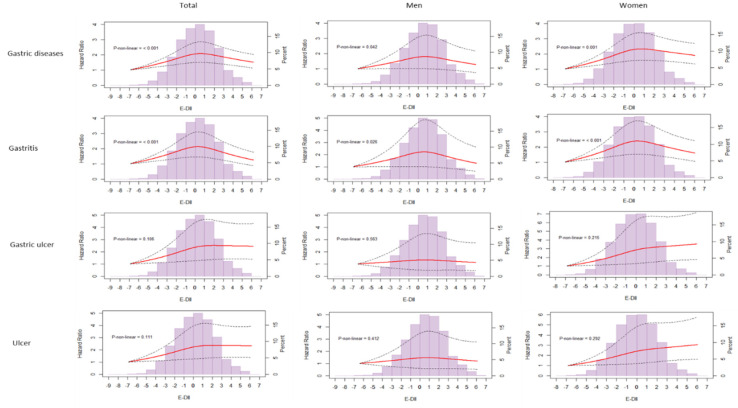
Dose-response analysis of the KoGES_HEXA using spline regression for the association between E-DII and gastric disease risk among the total population, men, and women. Solid lines illustrate point estimates and dashed lines represent 95% confidence intervals. Hazard ratios were calculated by the model adjusted for sex, age, education, smoking, drinking, physical activity, and BMI. The histograms show the distribution of E-DII levels in each corresponding group.

As shown in Figure 3, a significant dose–response association was observed for gastric ulcers and gastric and duodenal ulcers in the total cohort (*p* > 0.05). No significant results were found for gastritis or gastric disease.

## 4. Discussion

The results based on data from the KoGES_HEXA cohort were consistent with our hypothesis, confirming that higher E-DII scores were associated with higher gastric disease risk after adjusting for potential confounders such as sex, age, smoking, alcohol consumption, and energy. In addition, females showed a stronger and consistently statistically significant association between the E-DII and gastritis and gastric ulcer risk than what was observed in males. Moreover, postmenopausal females showed an increased risk of gastritis and gastric ulcers with increasing consumption of a pro-inflammatory diet.

The DII has evolved as a powerful tool for estimating an individual’s diet based on its inflammatory properties [35]. In over 40 construct validation studies conducted to examine associations with inflammatory markers, the DII has successfully predicted the levels of inflammatory markers such as interleukins, CRP, and tumor necrosis factor [36]. Pro-inflammatory diets, consisting largely of ultra-processed and fried foods, red and processed meat, dairy products, sweets and desserts, and refined grains, are associated with high levels of inflammation [37]. In contrast, anti-inflammatory diets rich in fruits and vegetables, flavonoids and antioxidants, whole grains and Omega−3 fats, and olive oil attenuate systemic inflammation [38]. Higher DII scores have been significantly associated with an increased risk of several health conditions, such as cancer, metabolic syndrome, cardiovascular diseases, diabetes, depression, rheumatoid arthritis, osteoarthritis, obesity, frailty, and overall and cause-specific mortality [39,40,41,42,43,44,45,46,47,48,49,50,51,52,53,54,55,56,57]. This evidence provides a good background for testing whether a pro-inflammatory diet, measured by the E-DII score, is associated with gastric disease risk.

To our knowledge, this is the first attempt to examine the association between the inflammatory potential of diet and the risk of gastric disease in the Korean population. The current study found a statistically significant association between the DII, E-DII, and gastric disease risk. According to a cross-sectional study with 574 participants in the Chinese population, high consumption of alcohol, meat, and coarse cereals was associated with an increased risk of chronic atrophic gastritis without *H. pylori* infection in males, although the dietary pattern was not associated with atrophic gastritis in females [58]. Similarly, ingestion of fried foods, barbecued foods, snacks, and very sweet, spicy, and salty foods from Western dietary patterns may activate innate immunity by producing excessive amounts of pro-inflammatory cytokines, resulting in chronic systemic or tissue-specific simmering inflammation in the stomach, gastric distension, and chronic or frequent stomach aches [59]. In patients with chronic gastritis, gastrointestinal symptoms were correlated with unhealthy dietary factors and food preferences, among which irregular mealtimes and consumption of salty and sweet foods were found to be the strongest risk factors [25]. From these studies, it is evident that dietary and lifestyle habits influence the risk of the progression of precancerous gastric lesions, leading to disease progression. It is also recommended that anti-inflammatory foods such as whole grains, fruits, vegetables, beans, legumes, and fish and other foods with abundant quantities of folate, flavonoids, and antioxidants be included in the diet [28,60].

Previous studies have shown that both sexes are susceptible to gastritis and ulcers, followed by chronic gastric cancer, but the results are inconsistent [61,62,63,64]. In our study, we found a significant association between the inflammatory potential of diet and the risk of gastric disease among females. In a longitudinal study of 54 cases of gastric carcinoma, higher rates of *H. pylori* infection, gastroesophageal reflux, and gastric inflammation were observed in females [64]. In contrast, in a meta-analysis, an elevated risk of gastric inflammation was observed in males compared to females, and long-term exposure to estrogen is considered a protective factor that inhibits gastric inflammation and gastric cancer [65]. Estrogen hormones regulate mucosal secretion, protect the mucosa from acid-induced damage, and regulate the growth and expression of gastric cells, thereby reducing the risk of gastric inflammation [66]. According to the Shanghai Women’s Health study of 73,442 study participants, progesterone and estrogen hormones are defensive components, and females with delayed menopause and long fertile years had a low incidence of gastritis and gastric carcinogenesis [67]. Future epidemiological studies with larger sample sizes are needed to confirm the sex differences in the association between diet-related inflammation and the incidence of gastritis.

In this study, we found a significant association between the E-DII and gastric ulcer risk among females. Based on data derived from the Korean National Health and Nutrition Examination Survey of 12,095 study participants, peptic ulcer disease risk was high in females compared to males after adjusting for potential confounders such as age, sex, BMI, education, aspirin use, stress, and metabolic disease [68]. Dietary patterns rich in fiber, vegetables, fruits, oatmeal, barley, peas, and lentils help to reduce cell damage and protect the mucosal lining of the stomach, thereby reducing the risk of peptic ulcers [69]. Similarly, females that consume a pro-inflammatory diet showed an increased prevalence of peptic ulcer disease with the presence of *H. pylori* infection [70]. Therefore, further large-scale studies are needed to confirm the association between sex differences and dietary patterns in peptic ulcer disease.

Our study observed a significant association between the E-DII and gastric disease risk among postmenopausal females. According to the National Center for Health Statistics in the United States, the hospitalization rate of most gastric ulcer patients aged 65 years has been reported [71]. Estrogen regulates human duodenal bicarbonate secretion and reduces the risk of ulcers and gastric disease in premenopausal females, and a decrease in the level of estrogen induces a depletion in the mucosal defense function [72]. Estrogen modulates vascular protection, accelerates the growth of endothelial cells, and safeguards the gastric mucosa against acid-induced injury [73]. A decrease in estrogen levels may result in ulceration of the gastric mucosa with severe inflammation and symptoms such as mood swings, depression, and irritation [74]. Further studies with larger sizes are needed to confirm the role of estrogens in gastric diseases.

In our study, fiber, green/black tea, flavan-3-ol, flavonols, nuts, spinach, lettuce, cucumber, carrot, and fruit (data not shown) were associated with a reduced risk of gastric disease. Several studies have shown that the intake of certain foods and nutrients is considered an important aspect of the origin and inhibition of gastric diseases [75,76,77,78]. Pro-inflammatory foods stimulate the digestive system and metabolism in the gastric glands to release higher concentrations of hydrochloric acid, which, in turn, increases inflammation of the stomach lining [75,76]. Flavonoids are gastro-protective agents that increase blood flow in the mucosa and stimulate the production of glycoproteins, glycolipids, and mucins in the gastric mucosa [77]. A case–control study with 299 cases and 433 healthy controls conducted in China found that green tea consumption produced a protective effect against chronic gastric disease with a dose–response relationship and reduced the risk of the progression of gastric cancer [78]. Similarly, in a Japanese study with 1696 study participants, the intake of green tea showed an inhibitory effect, and the polyphenols found in green tea acted as scavengers, inhibited the release of pro-inflammatory cytokines, and fought against gastric inflammation [79]. A high-fiber diet serves as an intermediate component, lowers the concentrations of gastric juices in the stomach, prevents erosion of the mucus membrane, and provides nourishment to the mucosal lining [80].

The current study has several strengths. It is the first to investigate the association between the inflammatory potential of diet and gastric disease risk in the Korean population. This cohort study, with large sample size, long-term follow-up, and inclusion of subjects free of gastric diseases at baseline, reduced the chance of recall bias and reverse causation. A third strength is that the population-based nature of the study increases the external validity and provides reliable results. In addition, a large number of Korean adults were included in the KoGES_HEXA cohort, which had high internal validity. Finally, the study findings can be used as clinical recommendations to address the importance of anti-inflammatory dietary patterns.

Despite its strengths, this study has several limitations that need to be mentioned. First, the study participants were recruited from health examination centers located in urban areas of Korea. Those willing to participate were potentially more health-conscious, leading to selection bias. Second, there were more females than males included; therefore, it might not represent the entire Korean population. Third, the diagnosis of gastritis was self-reported by the study subjects; so, errors may have been incurred. Fourth, although the FFQ is a common dietary assessment method, it contains a defined list of food items, and subjects may not have been able to report their food intake accurately or may have reported biased intake, as seen in the United States [81]. Fifth, the status of *H. pylori* infection, usage of non-steroidal anti-inflammatory drugs, and autoimmune chronic atrophic gastritis information were not included. Finally, the levels of inflammatory biomarkers such as IL-1, IL-4, IL-6, and IL-10 were not measured.

## 5. Conclusions

Diets with high pro-inflammatory properties estimated by the E-DII were associated with a higher risk of gastric disease incidence among Koreans, especially in females. We expect that the current study will contribute to the importance of recommending healthy diet consumption, raise awareness of pro-inflammatory diets, and provide recommendations and guidelines for gastric disease prevention and public health promotion.

## Figures and Tables

**Table 1:** Baseline characteristics of study subjects across quartiles of E-DII in KoGES_HEXA (*n* = 144,196).

Characteristics	E-DII Quartiles ^a^				*p* Value ^d^
	Q1 (*n* = 36,049)	Q2 (*n* = 36,049)	Q3 (*n* = 36,049)	Q4 (*n* = 36,049)	
E-DII score	−8.20 to −1.22	−1.22 to 0.23	0.23 to 1.61	1.61 to 6.36	
Energy (kcal/day) ^b^	1805.0 ± 593.5	1802.0 ± 520.6	1751.1 ± 481.7	1644.5 ± 459.1	<0.0001
Age (years) ^b^	52.9 ± 8.0	52.6 ± 8.2	53.7 ± 8.5	54.1 ± 8.8	<0.0001
BMI (Kg/m^2^) ^b^	24.3 ± 5.8	24.1 ± 5.1	23.9 ± 4.6	23.7 ± 5.2	<0.0001
<18.5 ^c^	584 (1.6)	649 (1.8)	687 (1.9)	814 (2.3)	<0.0001
18.5–<23	13,286 (36.7)	13,506 (37.5)	13,215 (36.7)	12,850 (35.6)	
23–25	9934 (27.6)	10,025 (27.8)	9973 (27.7)	9917 (27.5)	
>25	12,245 (34.1)	11,869 (32.9)	12,174 (33.8)	12,468 (34.6)	
Sex ^c^, Female	26,634 (73.9)	24,622 (68.3)	22,785 (63.2)	20,327 (56.4)	<0.0001
Male	9415 (26.1)	11,427 (31.7)	13,264 (36.8)	15,722 (43.6)	
Marital status ^c^, Married	31,851 (88.4)	31,933 (89.6)	31,805 (88.2)	31,010 (86.0)	<0.0001
Single/divorced/widowed	4198 (11.6)	4116 (11.4)	4244 (11.8)	5039 (14.0)	
Education ^c^, <Middle school	5125 (14.2)	5452 (15.1)	6315 (17.5)	7959 (22.1)	<0.0001
Middle~high school	20,494 (56.8)	19,705 (54.7)	19,564 (54.3)	19,409 (53.8)	
≥College	10,430 (29.0)	10,892 (30.2)	10,170 (28.2)	8681 (24.1)	
Smoking ^c^, Never	28,587 (79.3)	26,987 (74.9)	25,774 (71.5)	23,496 (65.2)	<0.0001
Past	4174 (11.6)	5028 (13.9)	5565 (15.4)	6339 (17.6)	
Current	3288 (9.1)	4034 (11.2)	4710 (13.1)	6214 (17.2)	
Drinking ^c^, Never	19,511 (54.1)	18,395 (51.0)	17,875 (49.6)	17,189 (47.7)	<0.0001
Past	1386 (3.8)	1330 (3.7)	1478 (4.1)	1672 (4.6)	
Current	15,152 (42.1)	16,324 (45.3)	16,696 (46.3)	17,188 (47.7)	
Physical activity ^e^, Irregular	14,401 (39.9)	16,572 (46.0)	18,143 (50.3)	20,174 (56.0)	<0.0001
Regular	21,648 (60.1)	19,477 (54.0)	17,906 (49.7)	15,875 (44.0)	
Menopausal status ^f^, Post	15,971 (60.1)	14,200 (57.7)	13,109 (57.5)	12,564 (61.8)	<0.0001
Pre	10,663 (39.9)	10,422 (42.3)	9676 (42.5)	7763 (38.2)	
Family history No	31,396 (87.1)	31,463 (87.3)	30,521 (84.7)	30,295 (84.0)	<0.0001
of cancer Yes	4673 (12.9)	4586 (12.7)	5528 (15.3)	5754 (16.0)	

^a^ E-DII is presented by quartiles at baseline, which divides the E-DII scores into four levels. Q1 represents the anti-inflammatory index, while Q4 represents the maximum pro-inflammatory index. ^b^ The data for continuous variables are presented as mean and standard deviation. ^c^ The data for categorical variables are presented as *n* (%) among all participants. ^d^ ANOVA and chi-square test was used to calculate *p* values for continuous and categorical variables, respectively. ^e^ Physical activity was measured based on whether the study participants regularly engaged in any sports until sweating. ^f^ The data for menopausal status were calculated only for women.

**Table 2:** Association between E-DII and gastric disease risk for all participants in cross-sectional analysis (*n* = 144,196) and prospective analysis (*n* = 142,755) of the KoGES_HEXA.

E-DII Quartiles ^a^	Cross-Sectional (Logistic Regression) ^b^				Prospective (Cox Proportional) ^c^			
	Cases/Total	Total	Men	Women	Cases/Total	Total	Men	Women
		Multivariate OR	Multivariate OR	Multivariate OR		Multivariate HR	Multivariate HR	Multivariate HR
**All Gastric Diseases**								
Q1	324/36,049	1.00	1.00	1.00	530/35,689	1.00	1.00	1.00
Q2	369/36,049	1.10 (0.97–1.25)	1.01 (0.79–1.28)	1.15 (0.99–1.33)	598/35,689	1.19 (1.06–1.34)	1.10 (0.86–1.42)	1.22 (1.06–1.39)
Q3	392/36,049	1.22 (1.07–1.40)	1.28 (1.00–1.65)	1.20 (1.02–1.41)	604/35,688	1.25 (1.11–1.41)	1.22 (0.96–1.55)	1.25 (1.09–1.44)
Q4	356/36,049	1.09 (0.94–1.26)	1.01 (0.77–1.32)	1.13 (0.95–1.34)	541/35,689	1.22 (1.08–1.38) *	1.15 (0.91–1.46)	1.27 (1.08–1.45) *
*p* for trend ^d^		0.20	0.14	0.19		0.01	0.21	0.01
*p* for interaction ^e^		0.23				0.10		
Continuous		1.01 (0.98–1.04)	0.99 (0.95–1.05)	1.02 (0.99–1.05)		1.02 (1.01–1.06) *	0.99 (0.93–1.01)	1.03 (1.01–1.06) *
**Gastritis**								
Q1	210/36,049	1.00	1.00	1.00	386/35,689	1.00	1.00	1.00
Q2	218/36,049	1.06 (0.93–1.22)	0.99 (0.74–1.34)	1.10 (0.93–1.31)	415/35,689	1.14 (0.99–1.32)	1.06 (0.78–1.45)	1.14 (0.96–1.35)
Q3	244/36,049	1.17 (1.01–1.39)	1.23 (0.91–1.69)	1.15 (0.96–1.39)	410/35,688	1.16 (1.01–1.34)	1.12 (0.84–1.50)	1.19 (1.02–1.39)
Q4	184/36,049	1.12 (0.94–1.27)	1.10 (0.79–1.54)	1.12 (0.91–1.36)	360/35,689	1.19 (1.04–1.37) *	1.17 (0.87–1.57)	1.19 (1.02–1.40) *
*p* for trend ^d^		0.13	0.16	0.27		0.01	0.29	0.03
*p* for interaction ^e^		0.21				0.84		
Continuous		1.02 (0.99–1.05)	1.00 (0.95–1.07)	1.02 (0.98–1.07)		1.02 (1.01–1.04) *	0.94 (0.90–0.98)	1.03 (1.01–1.06) *
**Gastric Ulcer**								
Q1	94/36,049	1.00	1.00	1.00	123/35,689	1.00	1.00	1.00
Q2	127/36,049	1.16 (0.90–1.49)	0.88 (0.58–1.33)	1.36 (0.99–1.87)	158/35,689	1.39 (1.1–1.76)	1.08 (0.71–1.63)	1.48 (1.11–1.96)
Q3	123/36,049	1.31 (0.99–1.70)	1.27 (0.83–1.94)	1.35 (0.96–1.90)	166/35,688	1.44 (1.13–1.83)	1.18 (0.77–1.81)	1.57 (1.18–2.09)
Q4	146/36,049	1.22 (0.92–1.61)	0.98 (0.61–1.56)	1.38 (0.97–1.97)	153/35,689	1.47 (1.16–1.85) *	1.23 (0.81–1.85)	1.68 (1.25–2.25) *
*p* for trend ^d^		0.15	0.32	0.14		0.01	0.08	0.01
*p* for interaction ^e^		0.31				0.15		
Continuous		1.03 (0.98–1.10)	1.02 (0.93–1.11)	1.05 (0.98–1.13)		1.02 (1.01–1.05) *	0.96 (0.93–1.01)	1.04 (1.01–1.15) *
**Duodenal Ulcer**								
Q1	20/36,049	1.00	1.00	1.00	21/35,689	1.00	1.00	1.00
Q2	24/36,049	0.86 (0.45–1.65)	0.90 (0.31–2.67)	0.84 (0.37–1.91)	25/35,689	1.21 (0.68–2.17)	1.85 (0.59–5.73)	1.05 (0.53–2.08)
Q3	25/36,049	1.54 (0.84–2.90)	1.37 (0.50–4.06)	1.63 (0.76–3.63)	28/35,688	1.36 (0.77–2.40)	1.88 (0.58–1.09)	1.07 (0.54–2.15)
Q4	26/36,049	0.98 (0.50–1.98)	0.92 (0.30–2.98)	1.00 (0.42–2.44)	28/35,689	1.38 (0.78–2.44)	2.30 (0.75–2.06)	1.31 (0.66–2.59)
*p* for trend ^d^		0.27	0.41	0.35		0.35	0.25	0.47
*p* for interaction ^e^		0.24				0.07		
Continuous		0.98 (0.86–1.12)	0.99 (0.81–1.24)	0.97 (0.82–1.15)		0.94 (0.89–0.97)	0.95 (0.81–1.09)	0.97 (0.72–0.99)
**Gastric and Duodenal Ulcer**								
Q1	114/36,049	1.00	1.00	1.00	144/35,689	1.00	1.00	1.00
Q2	151/36,049	1.11 (0.88–1.40)	0.88 (0.59–1.30)	1.27 (0.94–1.70)	183/35,689	1.35 (1.08–1.69)	1.19 (0.79–1.78)	1.42 (1.09–1.85)
Q3	148/36,049	1.33 (1.05–1.71)	1.28 (0.87–1.91)	1.38 (1.01–1.89)	194/35,688	1.45 (1.16–1.81)	1.32 (0.90–1.94)	1.50 (1.15–1.96)
Q4	172/36,049	1.19 (0.92–1.54)	0.98 (0.63–1.51)	1.33 (0.96–1.85)	181/35,689	1.46 (1.17–1.81) *	1.14 (0.78–1.69)	1.65 (1.26–2.17) *
*p* for trend ^d^		0.12	0.23	0.11		0.01	0.50	0.01
*p* for interaction ^e^		0.26				0.16		
Continuous		1.03 (0.98–1.09)	1.01 (0.93–1.10)	1.04 (0.98–1.11)		1.05 (1.01–1.06) *	1.01 (0.91–1.08)	1.06 (1.02–1.13) *

^a^ E-DII is presented by quartiles at baseline, which divides the E-DII scores into four levels, Q1 represents the anti-inflammatory index, while Q4 represents the maximum pro-inflammatory index. ^b^ Odds ratios (ORs) and 95% CI were calculated using a logistic regression model. ^c^ Hazard ratios (HRs) and 95% CI were calculated using the Cox proportional hazards model. Both models were adjusted for sex, age, education status, smoking, drinking, physical activity, and BMI. ^d^ *p* for trend was computed by assigning the median value for each quartile and treating it as a continuous variable. ^e^ *p* for interaction was calculated from the likelihood ratio test comparing the multivariate-adjusted model with and without the product terms of E-DII and sex. * Significant difference.

## Supplementary Materials

The following are available online at https://www.mdpi.com/article/10.3390/nu14132662/s1. Table S1: Distribution of food and nutrient components across quartiles of E-DII score in the KoGES_HEXA (*n* = 144,196); Table S2: Association between E-DII score and gastric disease risk in women, stratified by menopausal status; Table S3: Association between E-DII score and gastric disease risk, stratified by age category. Dietary assessment using SQ-FFQ and calculation of DII [82,83].
