# Supplementary material for: Association between the Dietary Inflammatory Index and Gastric Disease Risk: Findings from a Korean Population-Based Cohort Study
Source: Nutrients. 2022 Jun 27;14(13):2662. doi: 10.3390/nu14132662 (PMC9268659; doi:10.3390/nu14132662)
Supplement: Supplementary file 1 [file nutrients-14-02662-s001.zip › nutrients-1757795-supplementary.pdf]

SUPPLEMENTARY DATA

This file includes:

Supplementary text for Materials and Methods
Supplementary Tables S1, S2, S3

MATERIALS AND METHODS

Dietary assessment using SQ-FFQ and calculation of DII

Dietary food intake at baseline was evaluated using a semi-quantitative food frequency questionnaire (SQ-FFQ). A test of the validity of the SQ-FFQ was determined in our previous study [82]. Dietary data were measured at the beginning of the study once, regarding their past year intake. The study participants reported the daily intake amounts (g), portion sizes and frequency of consumption of 106 food and beverage items consumed over the course of one year. Portion sizes were calculated according to three replies: half a serving, a standard serving and three-half servings. The frequency of each food item was recorded based on nine replies: almost never, once per month, 2–3 times per month, 1–2 times per week, 3–4 times per week, 5–6 times per week, once per day, twice per day, and more than three times per day. Daily nutrient intakes and total calories were estimated using a food composition table [83].

An entire updated information on DII are available elsewhere [84]. Briefly, a total of 1943 research articles were screened for eligibility, analyzed and scored. Scoring for the 45 food parameters was based on their effects on the levels of these inflammatory biomarkers: CRP, IL-1, IL-4, IL-6 and TNF. The nutritional data used in the current study were determined from the Functional Ingredients Table (Rural Development Administration), Computer Aided Nutritional Analysis (The Korean Nutrition Society) and the U.S. Department of Agriculture. A global comparison database representing diet surveys from eleven countries were used as a comparative standard for each of the forty-five food parameters. Z-scores were determined by subtracting the standard global mean for each parameter from the amount reported and dividing by the global standard deviation. To minimize the effects of right-skewing, the z-scores were converted into proportion, doubled and then 1 was

subtracted from each to center the average score on zero. The resulting value was multiplied by the corresponding food parameter effect score, and these were then summed to obtain the overall DII score. The energy-adjusted DII (E-DII) score was calculated similarly, but using intakes calculated per 1000 kcal and the energy-adjusted global reference database was used to obtain the z-scores. A high E-DII score represents a more pro-inflammatory diet, and a low E-DII score represents an anti-inflammatory diet. In this study, 37 of the possible 45 food parameters were scored for DII development.

Measurement of covariates

In the present study, age, energy intake, BMI, were measured as continuous variables and sex, marital status, educational status, smoking, alcohol consumption, physical activity, menopausal status was measured as categorical variables. Gender was classified by biological sex namely male and female. Marital status was classified into two categories namely married and single or divorced or widowed. Educational status was divided into less than middle school, middle school to high school and college or higher. With respect to smoking, 'current' smokers are subjects who had smoked more than 400 cigarettes to date and continued to smoke at the time of the survey. 'Past' smokers are those who smoked more than 400 cigarettes but quit smoking. 'Never' smokers are those who had not smoked more than 400 cigarettes. As for drinking, those who had been drinking at the time of the survey were classified as 'current' drinkers, those who had been drinking but refraining at the time of the survey as 'past' drinkers, and those who never drank as 'never' drinkers. Regularity of physical activity was determined based on whether the subjects were exercising consistently in any sports to the point of sweating. Those who accomplish the criteria were considered as 'regular' in terms of physical activity, while those who did not accomplish the criteria were considered as 'irregular' in terms of physical activity. Menopausal status was divided into pre-menopause (presence of monthly menstrual cycle) and post-menopause (absence of monthly menstrual cycle). The family history of cancer was classified as follows (no, yes).

References

82. Ahn Y, Kwon E, Shim JE, Park MK, Joo Y, Kimm K, et al. Validation and reproducibility of food frequency questionnaire for Korean genome epidemiologic study. *European Journal of Clinical Nutrition.* 2007;61(12):1435-1441.

83. Institute. NRRD. Food Composition Table, 9th ed.; Rural Development Administration. National Rural Resources Department Institute: Suwon, Korea. *Available online:http://koreanfoodrdagokr/eng/fctFoodSrchEng/engMain(accessed on 17 March 2021).*

Table S1. Distribution of food and nutrient components across quartiles of E-DII score in the KoGES_HEXA (n=144,196)

Food and nutrient components[b]	Components quartiles[a]				P-value[c]
	Q1 (n=36,049)	Q2 (n=36,049)	Q3 (n=36,049)	Q4 (n=36,049)	
Cereals(g/day)	0.3 ± 2.4	0.4 ± 2.4	0.3 ± 2.5	0.4 ± 2.6	0.02
Nuts(g/day)	1.4 ± 3.6	1.4 ± 3.6	1.4 ± 3.5	1.4 ± 3.7	0.01
Eggs(g/day)	11.8 ± 15.9	11.6 ± 15.2	11.5 ± 15.3	11.5 ± 15.1	0.02
Spinach(g/day)	7.3 ± 13.6	7.2 ± 13.1	7.1 ± 13.1	7.0 ± 12.7	0.01
Carrot	3.1 ± 5.4	3.1 ± 5.4	3.1 ± 5.5	3.1 ± 5.5	0.02
Pork(g/day)	13.6 ± 20.1	13.5 ± 19.1	13.5 ± 19.4	13.7 ± 20.3	0.03
Chicken(g/day)	4.0 ± 7.3	4.0 ± 7.1	4.1 ± 8.3	4.1 ± 7.4	0.03
Beef(g/day)	2.1 ± 4.9	2.1 ± 5.3	2.1 ± 4.7	2.1 ± 5.3	0.50
Milk(g/day)	78.6 ± 112.0	78.6 ± 112.9	78.8 ± 112.6	77.6 ± 111.7	0.04
Coffee(g/day)	3.6 ± 3.1	3.6 ± 3.1	3.6 ± 3.1	3.6 ± 3.1	0.03
Carbonated beverages(g/day)	10.6 ± 43.9	10.3 ± 41.9	10.8 ± 45.3	10.7 ± 46.5	0.05
Vitamin B6 (mg/day)	2.4 ± 0.7	1.6 ± 0.3	1.2 ± 0.3	1.1 ± 0.3	<.0001
Carotene (μg/day)	4506.0 ± 2482.4	2347.7 ± 903.7	1566.1 ± 762.1	1291.0 ± 660.1	<.0001
Caffeine (g/day)	0.1 ± 0.1	0.1 ± 0.1	0.1 ± 0.1	0.1 ± 0.1	<.0001
Carbohydrate (g/day)	374.2 ± 93.5	320.7 ± 73.9	282.5 ± 70.4	266.1 ± 69.9	<.0001
Vitamin B12 (μg/day)	9.9 ± 5.9	6.0 ± 3.0	4.1 ± 2.4	3.3 ± 2.2	<.0001
Cholesterol (mg/day)	254.8 ± 144.7	174.2 ± 95.2	124.9 ± 80.9	106.7 ± 76.8	<.0001
Total fat (g/day)	40.7 ± 19.1	29.4 ± 13.2	21.8 ± 11.1	19.0 ± 10.6	<.0001
Fiber (g/day)	9.2 ± 3.2	5.9 ± 1.4	4.4 ± 1.4	3.8 ± 1.3	<.0001
Folic acid (μg/day)	363.1 ± 136.1	221.9 ± 54.0	158.6 ± 53.0	133.2 ± 48.6	<.0001

Garlic (g/day)	0.8 ± 1.6	0.3 ± 0.7	0.2 ± 0.5	0.2 ± 0.4	<.0001
Iron (mg/day)	15.6 ± 5.3	10.3 ± 2.3	7.7 ± 2.2	6.6 ± 2.0	<.0001
Magnesium (g/day)	96.3 ± 43.1	60.3 ± 21.0	43.0 ± 18.9	36.8 ± 17.7	<.0001
MUFA (g/day)	12.4 ± 7.8	8.6 ± 5.0	6.2 ± 4.2	5.3 ± 4.1	<.0001
Niacin (mg/day)	20.6 ± 6.3	15.0 ± 3.6	11.8 ± 3.3	10.5 ± 3.2	<.0001
n3 fatty acids (g/day)	0.6 ± 0.6	0.3 ± 0.3	0.2 ± 0.2	0.2 ± 0.2	<.0001
n6 fatty acids (g/day)	3.6 ± 2.3	2.6 ± 1.6	1.9 ± 1.3	1.7 ± 1.2	<.0001
Onion (g/day)	10.0 ± 8.5	6.9 ± 5.7	5.0 ± 4.6	4.3 ± 4.2	<.0001
Protein (g/day)	83.7 ± 26.2	61.6 ± 15.4	48.5 ± 13.7	43.3 ± 13.1	<.0001
PUFA (g/day)	7.5 ± 3.9	4.9 ± 2.4	3.4 ± 1.8	2.3 ± 1.4	<.0001
Riboflavin (mg/day)	1.4 ± 0.5	1.0 ± 0.2	0.7 ± 0.2	0.6 ± 0.2	<.0001
Saturated fat (g/day)	13.6 ± 7.5	9.8 ± 5.0	7.3 ± 4.2	6.3 ± 4.1	<.0001
Selenium (μg/day)	165.4 ± 47.0	140.6 ± 35.1	123.5 ± 32.2	117.2 ± 31.6	<.0001
Thiamin (mg/day)	1.4 ± 0.4	1.0 ± 0.3	0.8 ± 0.3	0.7 ± 0.3	<.0001
Trans fat (g/day)	0.6 ± 0.6	0.4 ± 0.4	0.3 ± 0.3	0.2 ± 0.2	<.0001
Vitamin A (mg/day)	879.6 ± 433.6	476.1 ± 158.4	321.1 ± 141.1	264.8 ± 123.9	<.0001
Vitamin C (mg/day)	188.2 ± 78.2	112.5 ± 36.0	74.5 ± 30.6	55.8 ± 26.0	<.0001
Vitamin D (μg/day)	5.3 ± 3.9	3.2 ± 2.0	2.2 ± 1.6	1.8 ± 1.4	<.0001
Vitamin E (mg/day)	13.2 ± 4.4	8.5 ± 2.1	6.1 ± 1.9	4.9 ± 1.7	<.0001
Zn (mg/day)	11.1 ± 4.4	8.2 ± 2.3	6.6 ± 1.9	5.9 ± 1.8	<.0001
Green/black tea (g/day)	105.7 ± 132.5	65.1 ± 96.8	37.4 ± 65.2	2.8 ± 18.9	<.0001
Flavan-3-ol (mg/day)	9388.6 ± 11730.9	5786.8 ± 8573.2	3324.4 ± 5770.1	254.4 ± 1673.6	<.0001
Flavones (mg/day)	58.7 ± 59.2	35.1 ± 34.3	23.0 ± 23.1	15.4 ± 17.9	<.0001
Flavonols (mg/day)	1437.0 ± 1642.5	882.8 ± 1192.7	512.9 ± 801.5	69.3 ± 233.1	<.0001
Flavanones (mg/day)	470.8 ± 624.6	300.6 ± 376.8	197.1 ± 250.3	132.2 ± 189.1	<.0001
Anthocyanidins (mg/day)	44.6 ± 35.4	27.6 ± 20.4	18.1 ± 14.7	13.6 ± 11.9	<.0001
Isoflavones (mg/day)	102.1 ± 112.5	76.1 ± 87.5	61.5 ± 75.8	55.3 ± 73.0	<.0001

[a]Food and nutrient components are presented by quartiles at the baseline, which divides the food and nutrient components into four levels, from Q1 to Q4. [b]The data for continuous variables are presented as mean and standard deviation. [c]ANOVA was used to calculate p values for continuous variables.

Table S2. Association between E-DII score and gastric disease risk in women, stratified by menopausal status

E-DII quartiles[a]	Case/Total	Pre-menopause<br>Multivariate HR[b]	Post-menopause<br>Multivariate HR[b]
**Gastritis**			
Q1	269/23144	1.00	1.00
Q2	313/23097	0.98 (0.96-1.11)	0.98 (0.84-1.03)
Q3	307/23105	0.99 (0.98-1.34)	1.05 (0.91-1.27)
Q4	270/23142	1.02 (0.99-1.39)	1.19 (1.15-1.39)*
P for trend[c]		0.25	0.01
P for interaction[d]		0.21	
Continuous		1.06 (0.99-1.10)	1.01 (0.98-1.09)
**Gastric Ulcer**			
Q1	76/23144	1.00	1.00
Q2	105/23097	0.76 (0.64-1.10)	1.20 (1.11-1.29)
Q3	104/23105	0.84 (0.78-1.06)	1.25 (1.17-1.34)
Q4	108/23142	1.03 (0.99-1.01)	1.32 (1.27-1.51)*
P for trend[c]		0.10	0.01
P for interaction[d]		0.11	
Continuous		1.03 (0.98-1.10)	1.11 (1.07-1.16)*
**Duodenal Ulcer**			
Q1	17/23144	1.00	1.00
Q2	13/23097	0.60 (0.52-1.06)	0.75 (0.66-0.91)
Q3	16/23105	0.71 (0.69-1.01)	0.92 (0.85-1.06)
Q4	18/23142	0.88 (0.77-1.01)	1.01 (0.91-1.27)
P for trend[c]		0.37	0.11
P for interaction[d]		0.19	
Continuous		1.06 (0.98-1.09)	0.95 (0.67-1.16)
**Gastric & duodenal ulcer**			
Q1	93/23144	1.00	1.00
Q2	118/23097	0.64 (0.48-1.15)	0.95 (0.83-1.15)
Q3	120/23105	0.78 (0.75-1.10)	1.18 (1.12-1.37)
Q4	126/23142	0.82 (0.65-1.08)	1.20 (1.13-1.45)*
P for trend[c]		0.14	0.02
P for interaction[d]		0.22	
Continuous		1.04 (1.00-1.07)	0.96 (0.78-1.13)

[a]E-DII is presented by quartiles at the baseline, which divides the E-DII scores into four levels, Q1 represents the anti-inflammatory index, while Q4 represents the maximum pro-inflammatory index. [b]Hazard Ratios (HRs) and 95% CI was calculated using Cox proportional hazard model. The model was adjusted for sex, age, education status, smoking, drinking, physical activity and BMI. [c]P for trend was computed by assigning the median value for each quartile and treating as a continuous variable. [d]P for interaction was calculated from the likelihood ratio test comparing the multivariate adjusted model with and without the product terms of E-DII and sex. *Significant difference $P < 0.05$.

Table S3. Association between E-DII score and gastric disease risk, stratified by age category

E-DII by age groups	Case/Total	Total	Men	Women
		Multivariate HR[a]	Multivariate HR[a]	Multivariate HR[a]
**Gastritis**				
≤ 65	1448/130315	0.95 (0.91-1.10)	0.74 (0.60-1.02)	1.03 (0.85-1.27)
> 65	123/12440	1.07 (1.03-1.24)*	0.71 (0.62-1.04)	1.13 (1.05-1.26)*
P for trend[a]		0.01	0.19	0.02
P for interaction[b]		0.16		
Continuous				
≤ 65		0.69 (0.60-1.11)	0.71 (0.59-1.02)	1.01 (0.95-1.02)
> 65		1.02 (1.01-1.15)*	0.67 (0.61-1.05)	1.02 (1.01-1.15)*
**Gastric Ulcer**				
≤ 65	551/130315	1.01 (0.89-1.15)	0.75 (0.62-1.02)	0.95 (0.71-1.25)
> 65	49/12440	1.21 (1.07-1.34)*	0.88 (0.72-1.10)	1.02 (1.01-1.14)*
P for trend[c]		0.01	0.15	0.02
P for interaction[d]		0.11		
Continuous				
≤ 65		0.98 (0.87-1.09)	0.73 (0.63-1.06)	0.89 (0.65-1.10)
> 65		1.07 (1.02-1.23)*	0.74 (0.70-1.01)	1.02 (1.01-1.10)*
**Duodenal Ulcer**				
≤ 65	95/130315	0.91 (0.81-1.20)	0.72 (0.60-1.02)	1.01 (0.76-1.27)
> 65	7/12440	0.98 (0.73-1.25)	0.86 (0.71-1.05)	1.07 (0.89-1.35)
P for trend[c]		0.24	0.23	0.09
P for interaction[d]		0.06		
Continuous				
≤ 65		0.87 (0.71-1.10)	0.71 (0.62-1.01)	0.90 (0.75-0.97)
> 65		1.00 (0.76-1.24)	0.76 (0.65-1.01)	1.02 (0.90-1.36)
**Gastric & duodenal ulcer**				
≤ 65	646/130315	0.80 (0.72-1.03)	1.14 (1.09-1.27)	1.10 (0.95-1.26)
> 65	56/12440	1.10 (1.02-1.09)*	0.89 (0.72-1.10)	1.08 (1.01-1.15)*
P for trend[c]		0.01	0.27	0.01
P for interaction[d]		1.10		
Continuous				
≤ 65		0.76 (0.70-1.04)	1.01 (0.89-1.15)	1.04 (0.86-1.36)
> 65		1.04 (1.01-1.09)*	0.82 (0.66-1.01)	1.05 (1.02-1.09)*

[a]Hazard Ratios (HRs) and 95% CI was calculated using Cox proportional hazard model. The model was adjusted for sex, age, education status, smoking, drinking, physical activity and BMI. [c]P for trend was computed by assigning the median value for each quartile and treating as a continuous variable. [d]P for interaction was calculated from the likelihood ratio test comparing the multivariate adjusted model with and without the product terms of E-DII and sex. *Significant difference $P < 0.05$.
